# Missed detection of significant positive and negative shifts in gentamicin assay: implications for routine laboratory quality practices

**DOI:** 10.11613/BM.2018.010705

**Published:** 2017-11-24

**Authors:** Gus Koerbin, Jiakai Liu, Alex Eigenstetter, Chin Hon Tan, Tony Badrick, Tze Ping Loh

**Affiliations:** 1 New South Wales Health Pathology, Chatswood, NSW 2067, Australia; 2 Department of Biomedical Science, University of Canberra, Canberra, Australia; 3 Department of Industrial and Systems Engineering, National University of Singapore, Singapore; 4 Faculty of Health Sciences and Medicine, Bond University, Queensland, Australia; 5 Department of Laboratory Medicine, National University Hospital, Singapore; 6 Biomedical Institute for Global Health Research and Technology, National University of Singapore, Singapore

**Keywords:** error, moving average, bias, quality control, quality assurance

## Abstract

**Introduction:**

A product recall was issued for the Roche/Hitachi Cobas Gentamicin II assays on 25^th^ May 2016 in Australia, after a 15 - 20% positive analytical shift was discovered. Laboratories were advised to employ the Thermo Fisher Gentamicin assay as an alternative. Following the reintroduction of the revised assay on 12^th^ September 2016, a second reagent recall was made on 20^th^ March 2017 after the discovery of a 20% negative analytical shift due to erroneous instrument adjustment factor.

**Materials and methods:**

The practices of an index laboratory were examined to determine how the analytical shifts evaded detection by routine internal quality control (IQC) and external quality assurance (EQA) systems. The ability of the patient result-based approaches, including moving average (MovAvg) and moving sum of outliers (MovSO) approaches in detecting these shifts were examined.

**Results:**

Internal quality control data of the index laboratory were acceptable prior to the product recall. The practice of adjusting IQC target following a change in assay method resulted in the missed negative shift when the revised Roche assay was reintroduced. While the EQA data of the Roche subgroup showed clear negative bias relative to other laboratory methods, the results were considered as possible ‘matrix effect’. The MovAvg method detected the positive shift before the product recall. The MovSO did not detect the negative shift in the index laboratory but did so in another laboratory 5 days before the second product recall.

**Conclusions:**

There are gaps in current laboratory quality practices that leave room for analytical errors to evade detection.

## Introduction

Laboratory analytical performance (precision and accuracy) is monitored routinely by a combination of internal quality control (IQC) and external quality assurance (EQA) activities. The IQC system involves periodic measurement of a set of QC samples at different concentrations and comparing these results against predefined control limits. It is aimed at providing a snapshot of the assay performance at the time of measurement to assist in the decision to make available results produced by the assay. On the other hand, a set of EQA materials is circulated and measured by a group of participating laboratories to promote transportability of results. It is important to note that IQC and EQA are not generally designed to monitor trueness.

Despite these quality systems, clinically significant changes in analytical performance can occur and yet evade detection by the laboratory. This may be due to the episodic nature of these quality systems, the use of inappropriate QC sample concentrations, the use of underpowered statistical techniques, or the presence of so called matrix effects in the materials used in IQC or EQA that may not fully represent the patient samples (*1*-*4*). Such significant shift or drift in analytical performance can occur during reagent lot change, and can cause an assay to report falsely high or low results.

Gentamicin is an aminoglycoside antibiotic commonly used for treatment of bacterial infections. It has a narrow therapeutic range. When the serum gentamicin concentration is within the toxicity range, it increases the risk of adverse effects such as impaired hearing and renal function. The renal impairment is generally reversible while hearing impairment can be permanent (*5*). A product recall was issued for the Roche/Hitachi Cobas Gentamicin II assays (Roche Diagnostics, Mannheim, Germany) on 25^th^ May 2016 in Australia, after a 15 - 20% positive analytical shift was reported (*6*).

In this report, we reviewed the practices of an index laboratory to examine how this analytical shift can evade detection by routine IQC and EQA systems in the Roche/Hitachi Cobas Gentamicin II assays, and examined the ability of the patient result-based approaches, including moving average (MovAvg) and the more recently described moving sum of outliers (MovSO) approaches in detecting this shift (*2*, *7*).

## Materials and methods

### GENT2 product recalls

The Roche Cobas GENT2 assay (Roche Diagnostics, Mannheim, Germany) is a one-step competitive kinetic interaction of microparticles in a solution immunoassay, and has been in use in the index laboratory since 2013. On 25^th^ May 2016, Roche issued an advisory to their GENT2 assay users of an increased recovery of 15 - 20% in patient results and initiated a reagent recall (for Cobas 701/702: part number: 05841291190, reagent lot numbers: 119167, 611783, 617624; for Cobas 501/502: part number 04490843190, reagent lot numbers: 119166, 611780, 617623). The assay was reinstated September 2016 with a recommended instrument adjustment factor of 0.8 to account for ‘calibrator matrix issue’. On 20^th^ Mar 2017, another product recall for the Roche GENT2 assay was issued by the Therapeutic Goods Administration (Australia) for erroneous instrument adjustment factor that was inappropriately low, leading to under-recovery of patient results. The sequence of events involving the analytical product recalls is summarized in Table 1. Of note, assay performance verification and comparison exercises were performed internally by the laboratory prior to routine implementation in compliance with the regulations of National Association of Testing Authorities, Australia.

**Table 1:** Sequence of events leading to the discovery of the analytical shift in the gentamicin assay at the laboratory reviewed

**Date**	**Event**
17^th^ May 2016	Roche advised of stock supply issues with GENT2 assay
25^th^ May 2016	Roche advised of increased recovery of patient results and reagent recall. Advised use of alternate assay.
31^st^ May 2016	Cessation of Roche assay
1^st^ Jun 2016	Switched to Thermo Fisher Gentamicin reagent
12^th^ Sep 2016	Roche advised GENT2 assay now available again but with 0.8 instrument adjustment factor due to ‘calibrator matrix issue’
20^th^ Oct 2016	Revised Roche assay with instrument adjustment factor of 0.8 reintroduced into clinical service
20^th^ Mar 2017	Therapeutic Goods Administration (Australia) recalled Roche GENT2 assay due to decreased patient recovery (Reference RC-2017-RN-00374-1). Instrument adjustment factor of 0.8 recommended by Roche was inappropriately low and laboratory was advised to reset instrument adjustment factor to 1.
24^th^ Mar 2017	Laboratory decided to reintroduce the Thermo Fisher Gentamicin reagent

### Missed detection by internal quality control procedure

At the index laboratory, the Chemtrak QC levels 1 and 3 (Thermo Fisher, San Jose, USA) for gentamicin were used. The targets for the QC material were established internally by the laboratory (for level 1: target 6.76 (standard deviation (SD) 0.21) mg/L; for level 3: target 1.56 (SD 0.06) mg/L). The mean values for the IQC samples used in the control chart are verified with each reagent/IQC lot change by measuring several replicates of IQC samples. This was performed most recently in January 2016 when there was an IQC lot change. The mean (target) value is changed, if the average result of the IQC samples using the new lot differs significantly (larger than the allowable limit of performance set by the Royal College of Pathologists of Australasia Quality Assurance Programme (RCPAQAP)) from the in-use reagent lot and the EQA results are acceptable. This procedure is undertaken to account for any lot-specific changes that may affect measurement of IQC materials secondary to matrix-related (commutability) issues. The assay capability (as determined by sigma metrics) is continuously monitored and the IQC control limits may be widened and reduced depending on the performance (standard deviation of IQC samples) of the assay.

**Figure 1:**
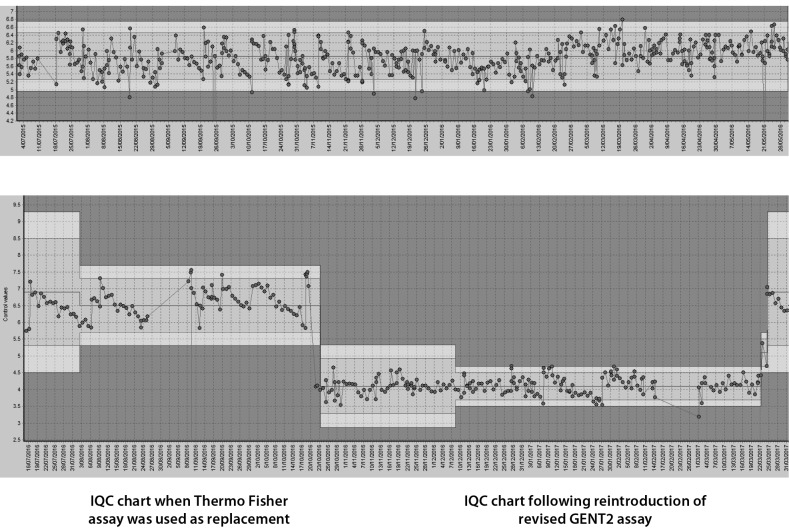
Internal quality control (IQC) data of the index laboratory. The upper panel shows the IQC chart showing acceptable results up to the 1^st^ Roche GENT2 reagent recall secondary to a 15 - 20% positive bias. The lower panel shows the IQC data during the use of the revised Roche assay with a 20% negative bias. The areas shaded light grey and grey represent 2 - 3 standard deviations and > 3 standard deviations, respectively.

During the time before the first reagent recall (*i.e.* prior to 25^th^ May 2016), the IQC did not detect any significant analytical shift, defined as any violation of the 2:2S, 1:3S or 4:1S rules (Figure 1, upper panel). On the other hand, when the laboratory reintroduced the revised GENT2 assay with an instrument adjustment factor of 0.8, there were indications of negative bias with multiple lower control limit violation (Figure 1, lower panel). However, because the index laboratory was using narrower control limits that were adapted from the Thermo Fisher assay while monitoring the capability of the revised GENT2 assay before deciding whether to widen them, these violations were placed on watching brief.

### Missed detection by external quality assurance programme

**Figure 2:**
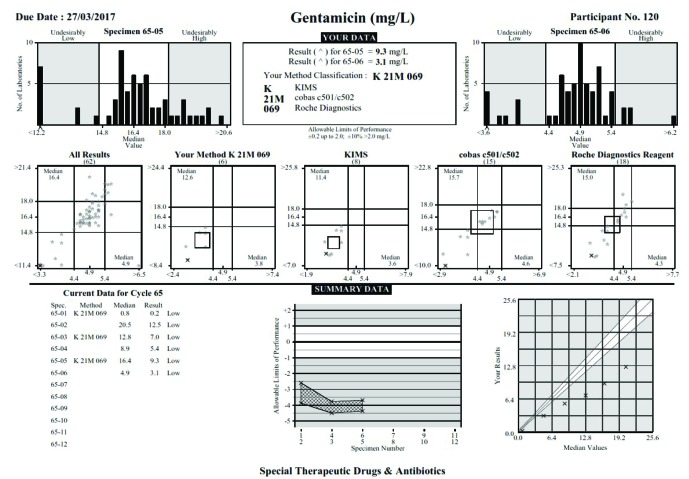
Analytical shift missed by the external quality assurance programme before the second reagent recall secondary to the presence of a negative bias, as the peer laboratories against which the index laboratory was compared were similarly using the affected reagents.

In the RCPAQAP, the peer performance of gentamicin assays is compared against the median results of the method subgroups. For the index laboratory, the gentamicin assay performed within acceptable limits during the affected period when compared to the other peer laboratories using the same affected reagents (Figure 2). However, the Roche Cobas subgroup showed a clear bias when compared to other laboratory methods. Owing to commutability considerations, it is probable that the laboratories using the affected reagent lot only interpreted their results using their peer group results and did not consider their difference to other laboratory methods as significant. Similarly, there was an excessive negative bias detected in the Roche peer subgroup following the change to the revised assay.

### Use of ‘moving statistics’ techniques for analytical shift detection

Given the above limitations of the routine quality systems, the ability of ‘moving statistics’ such as the MovAvg and the MovSO techniques in detecting the analytical shifts was examined. For this study, laboratory results for gentamicin between 1^st^ January 2015 and 5^th^ April 2017 were extracted from the laboratory information system.

#### Moving average

The MovAvg control chart was constructed as previously described (*2*). Briefly, laboratory results between 1^st^ January 2015 and 30^th^ June 2015 were taken as the analytically stable period, as evidenced by stable IQC and EQA performances. The data were used to calculate the population mean and standard deviation. Next we calculated the moving average of the block for the data from 1^st^ July 2015 to 5^th^ April 2017. A truncation limit of 5.0 mg/L was applied, which removed 4.4% of the overall data. The truncation limit was applied to remove extreme values (‘outliers’), which may skew the moving average. The removal of 4.4% of data using the truncation limit is within the recommended outlier removal as a proportion of the overall data of 20% (*8*). The upper and lower control limits were set as population mean ± 3 × 
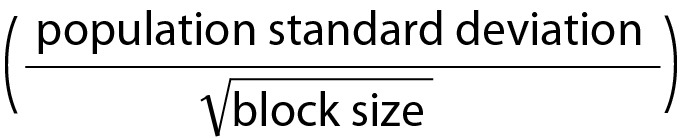
, respectively.

A block size of 100 was used for all the calculations in this study. A result that is below the detection limit (*i.e.* < 0.4 mg/L) was considered as 0.2 mg/L for the purpose of calculating the moving average. This assumption is acceptable in this scenario since at shift of 15 - 20% is too small to trigger the control limits. A MovAvg control chart was constructed by applying the population mean and control limits on the remaining gentamicin data from 1^st^ July 2015 to 5^th^ April 2017. An analytical error is detected when the moving average violates any of the control limits.

#### Moving sum of outliers

The MovSO control chart was constructed as recently described (*7*). Briefly, laboratory results from the stable period, as defined above, were used to derive the mean and SD of the number of outlier results, which was defined as any laboratory results that was above 1.0 mg/L (the cut-off value used by the laboratory). The upper and lower control limits were set as mean ± 3 × SD, respectively. A MovSO control chart was constructed by applying the mean and control limits on the remaining gentamicin data from 1^st^ July 2015 to 5^th^ April 2017. An analytical error is detected when the moving average violates any of the control limits. To confirm the findings, the same moving statistic techniques were applied to another laboratory (laboratory W) that was also using the Roche GENT2 assay within the same pathology network.

## Results

**Figure 3:**
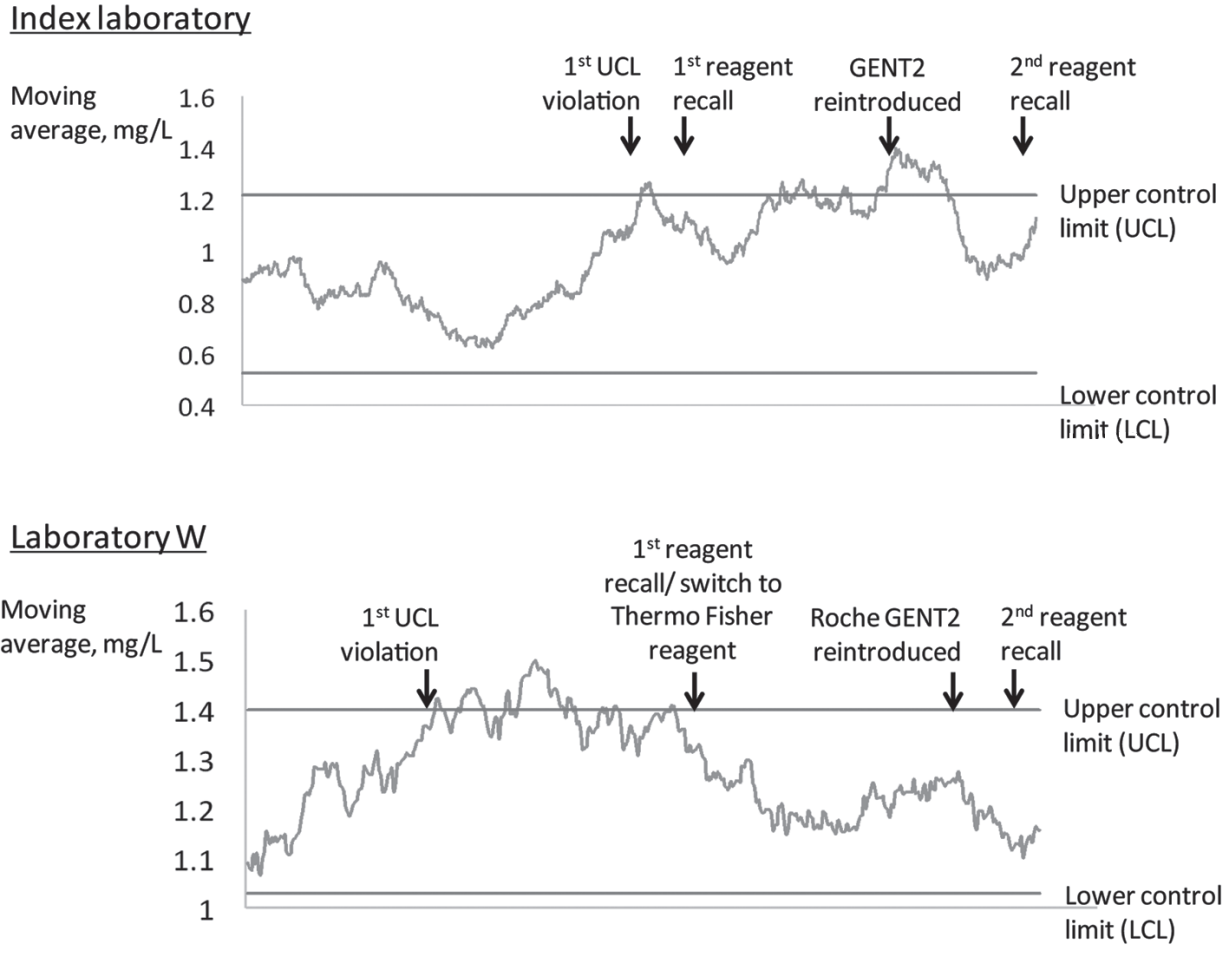
Moving average control chart of gentamicin from 1st July 2015 to 5th April 2017 for the index laboratory (upper panel) and laboratory W (lower panel). The horizontal lines represent the upper and lower control limits, respectively.

**Figure 4:**
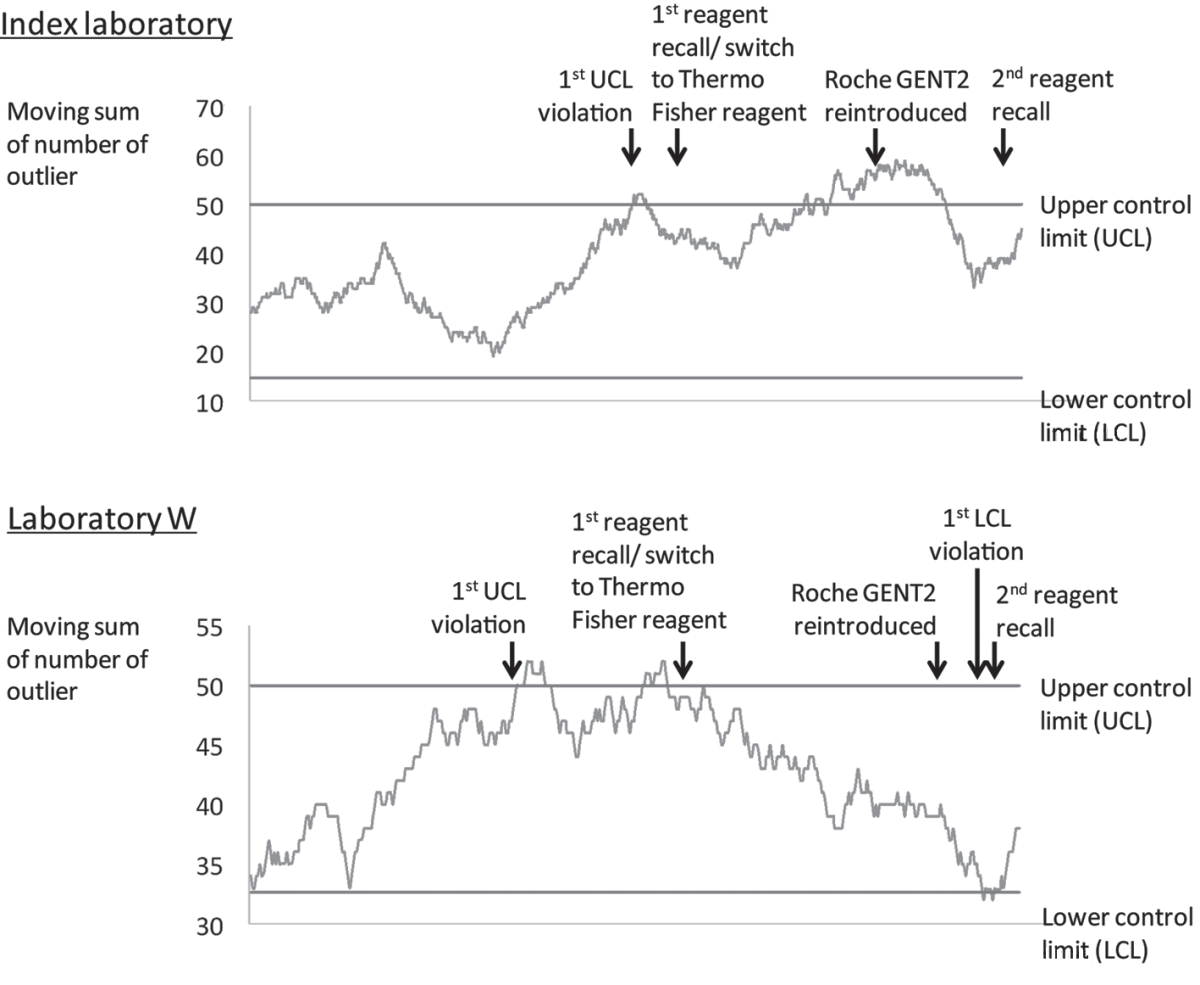
Moving sum of outlier control chart of gentamicin from 1st July 2015 to 5th April 2017 for the index laboratory (Panel A) and laboratory W (Panel B). A result of > 5.0 mg/L was considerd as an outlier. The horizontal lines represented the upper and lower control limits, respectively.

The results of the moving statistics are shown in Figures 3 and 4. The MovAvg technique first detected a positive shift in patient results on 20^th^ March 2016 while the MovSO detected a positive shift seven days earlier on 13^th^ March 2016. The MovAvg detected a positive bias on 13^th^ November 2015 at laboratory W. On the other hand, the MovSO detected a positive bias and a negative bias on 9^th^ January 2016 and 15^th^ March 2017 at laboratory W, respectively (Figures 3 and 4).

### Clinical impact

Additionally, consultation with both adult and child physicians was undertaken facilitated by the Clinical Excellence Commission, New South Wales, Australia. A retrospective clinical review was undertaken for both children and adults. The retrospective clinical review was for 12 months. The number of adults was restricted to those with multiple measurements within the episode of hospitalisation. The children retrospective review was based on the result concentration between 1.2 and 2.0 mg/L. Whilst there was a number of patients that fell into these categories, to date the physicians have reported no adverse effects.

## Discussion

There is accumulating evidence to show that the traditional quality control systems in routinely laboratory practice are inadequate to detect error in the increasingly complex testing environment (*4*, *7*). This is further confounded by the use of artificial IQC and EQA sample materials that may not fully represent patient samples. Laboratory staff often mistake shifts in a method group median as a matrix effect when it may represent a true bias in a method caused by a change in calibrator value or reagent reformulation. Relative changes in bias between different method subgroups should always be investigated particularly with assays that have been used for long periods. If the laboratory incorrectly assumes this shift is a matrix effect they may introduce post analytical factors such as the adjustment of IQC control chart mean / target value or EQA peer group comparison by median / mean. As illustrated in this report, these can have the unintended consequence of confounding the interpretation of the quality control data and missing an error the quality systems was designed to detect. Of note, there were 10 IQC rule violations prior to the first product recall but this rule was generally considered a ‘warning’ flag by the index laboratory and they ‘self-resolved’ upon further testing. This represents another missed opportunity for earlier error detection.

On the other hand, the use of moving statistics is an important tool to improve error detection in modern laboratories. They have been shown to out-perform traditional IQC approaches in detecting analytical shift, even at low analyte concentrations (*2*, *7*). The MovAvg technique is suitable for detecting large analytical shifts, whereas the MovSO is particularly suitable for detecting critical shifts at low concentrations that affects the classification of patient results (*7*). In this instance, the MovSO technique had very comparable performance to the MovAvg technique.

The main advantage of using moving statistics is that the analytical performance is monitored by actual patient results generated from clinical samples that the analytical system is designed to measure. However, these statistical techniques remain unfamiliar to many laboratory practitioners. Another barrier to practice is the relatively limited laboratory information systems that may not allow easy extraction and manipulation of patient results for the purpose of constructing these control charts.

Importantly, the moving statistics did not detect the 20% negative bias associated with the erroneous instrument adjustment factor of 0.8 that triggered the second reagent recall, except the MovSO when applied on laboratory W. This may be explained by the relatively wide control limits compared to the analytical shift. For example, the three standard deviations represented 40% and 50% of the mean (or central value) of the MovAvg and MovSO control charts of the index laboratory, respectively; and 15% and 21% of Laboratory W, respectively. This underscores the fact that the performance of the moving statistics is highly dependent on the variability of the underlying population being monitored since the patient and the analytical method contribute any observed variation (*8*, *9*). For a therapeutic drug monitoring test, the variability of the population result is likely to be high since it is not a physiologically tightly controlled substance. Additionally, there is a lag effect of using moving statistics, where an analytical shift needs to affect a relatively large number of patient results before the moving statistics can violate the control limit. One possible way to overcome this limitation may be to pool the laboratory data from multiple sites to increase the number of results. However, this is limited by the need for the different laboratories to be using the same reagent lot to maximize the statistical power.

This report illustrated the short coming of some of the common laboratory quality systems in detecting an analytical shift and also provided evidence that additional patient-based laboratory statistics is needed to optimise the risk management of laboratory errors. EQA providers have a role in this challenge by providing simple tools that allow a laboratory to identify significant changes in lot calibration by a manufacturer relative to other manufacturers or same manufacturer lots. However few EQA providers do that currently.
